# Resignations and Changes

**Published:** 1880-07-20

**Authors:** 


### EDITORIAL AND MISCELLANEOUS.

### *RESIGNATIONS AND CHANGES.*

*Dr. Lindsay Johnson* has resigned his place as Demonstrator of Anatomy and Lecturer on Minor Surgery in the Southern Medical College.

*Dr. James A. Gray* resigned his position in the Atlanta Medical College (the old school) and has been appointed Demonstrator of Anatomy and Auxiliary Professor of the "Principles and Practice of Surgery" in the Southern Medical College (the new school). Dr. Gray is able and well qualified for the duties assigned him.

*Dr. John Thad. Johnson* resigned his professorship in the old school and has accepted the Chair of the "Principles and Practice of Surgery" in the Southern Medical College, Atlanta. Dr. Johnson was for many years Dean of the Faculty and Prof. of Anatomy in the other school. He is an able lecturer and eminently fitted for the Chair which he has chosen in the new school.

*Dr. G. G. Crawford* will continue as heretofore in the chair of "Operative and Clinical Surgery." He is a fine operator.

*Dr. G. G. Roy* has been elected to the Chair of "Materia Medica" in place of Dr. G. M. McDowell, resigned. He is competent and will devote himself with zeal and energy to the position assigned him.

